# Bta-miR-376a Targeting *KLF15* Interferes with Adipogenesis Signaling Pathway to Promote Differentiation of Qinchuan Beef Cattle Preadipocytes

**DOI:** 10.3390/ani10122362

**Published:** 2020-12-10

**Authors:** Xingyi Chen, Sayed Haidar Abbas Raza, Gong Cheng, Xinhao Ma, Jianfang Wang, Linsen Zan

**Affiliations:** 1 College of Animal Science and Technology, Northwest A&F University, Yangling 712100, China; chenxingyi@nwafu.edu.cn (X.C.); haiderraza110@nwafu.edu.cn (S.H.A.R.); chenggong@nwafu.edu.cn (G.C.); MaXinhao@nwafu.edu.cn (X.M.); jfwang@nwsuaf.edu.cn (J.W.); 2 National Beef Cattle Improvement Center, Northwest A&F University, Yangling 712100, China

**Keywords:** Bta-miR-376a, KLF15, proliferation, adipocyte differentiation, beef quality

## Abstract

**Simple Summary:**

Our study demonstrated that bta-miR-376a negatively regulates adipocyte proliferation and differentiation by targeting the adipogenesis signaling pathway and is thus a novel regulator of development in Qinchuan beef cattle. These findings contribute to a better understanding of adipogenesis regulated by miRNA and provide an important reference for studies on beef intramuscular fat.

**Abstract:**

Intramuscular fat (IMF) is a quality index associated with the taste and juiciness of meat. The deposition of IMF is affected by genetic and non-genetic factors, such as age, slaughter location, gender of the animal, and diet. Micro-ribonucleic acids (miRNA) are transcriptional regulators involved in adipogenesis, but the specific role of miR-376a in regulation of bovine adipocytes remains unknown. Our findings indicated that miR-376a was a potential negative regulator of bovine adipocyte differentiation. A bta-miR-376a mimic inhibited mRNA and protein expression of the marker genes, *CDK1*, *CDK2*, *PCNA*, *C/EBPα*, *FAS*, and *PPAR γ*, and significantly reduced ratios (%) of S-phase cells, the number of cells stained with 5-ethynyl-2′-deoxyuridine, and adipocyte proliferation. Oil red O staining and triglyceride content analysis also confirmed that bta-miR-376a was involved in adipocyte differentiation. Luciferase activities confirmed that Krüppel-like transcription factor 15 (KLF15) was a direct target gene of bta-miR-376a, and that KLF15 was a key transcription factor in adipogenesis. Therefore, bta-miR-376a might be a target for increasing beef IMF.

## 1. Introduction

With the continuous growth of the world’s population and the improvement of living standards, insufficient production quality and quantity of animal-derived protein products have become the main problems facing world animal husbandry development. However, the study of fat deposition of Qinchuan cattle, a Chinese cattle breed which is famous for its fine texture of meat and relatively slow growth, will provide a new basis and ideas for beef cattle breed improvement, meat quality, and beef production [1].

The adipose tissue exerts a profound impact on physiology and pathophysiology [2]. It comprises various types of adipocytes, participates in the regulation of physiological and metabolic processes, and stores energy [3]. Adipose tissues play an important role in determination of meat quality. The main types of fat in mammals are intermuscular, intramuscular, subcutaneous, and peripheral. Among them, intramuscular fat (IMF) content is particularly important as a factor affecting the quality of beef: moderate content and uniform distribution of marbling can render meat tasty and juicy, whereas a low-fat content renders meat dry and dull [4]. However, increasing the amount of IMF without increasing the reserves of the other three types of fat is difficult [5]. Lipogenesis is a complex biological process that is closely related to energy and basic metabolism, and fat deposition involves a series of processes, including the proliferation, differentiation, and maturation of preadipocytes [6,7,8]. Adipogenesis is tightly controlled by a cascade of transcription factors [9]. MicroRNAs mediate gene silencing by guiding argonaute (Ago) protein to the target site of 3’UTR of mRNA, which plays an important role in gene regulation at the post-transcriptional level [10]. Most miRNAs exhibit high sequence conservation and tissue specificity [11]. MicroRNAs are involved in the regulation of adipocyte differentiation mainly via the following two mechanisms [12]: One mechanism involves signal pathways associated with adipocyte differentiation, including PPAR, MAPK, cAMP/PKA, and Wnt, whereas the other mechanism involves transcription factors such as PPARγ and C/EBP family members that regulate the expression of adipogenic genes such as *FABP4*, *FASN*, *GLUT4*, and other genes affect the differentiation of adipogenesis [3,6]. The miRNA miR-27a may play a key role in the differentiation of 3T3-L1 preadipocytes by inhibiting PPARγ [13]. MicroRNA-9 targets PNPLA3 through the AMPK pathway, thus inhibiting the differentiation of adipocytes in 3T3-L1 cells [14]. However, the effects of miRNAs related to the function and mechanism of adipogenesis require further investigation. A novel retinal preference for miR-376a, determined using a miR-microarray with altered expression in retinal diseases, has helped to elucidate the function of miRNA in normal and pathological retinas [15]. Current interest in miR-376a primarily focuses on cancer treatment [16,17,18]. The proliferation of osteosarcoma tumor cells is significantly inhibited by miR-127-3p and miR-376a-3p mimics [19]. The overexpression of miR-376a-3p inhibits the development of human umbilical vascular epithelial cells (HUVEC) by downregulating NRIP1 [20]. However, the mechanism by which miR-376a participates in the regulation of adipocyte differentiation remains unknown. Five miRNAs are differentially expressed with dynamic expression profiles in diabetic rat models of bone healing compared with controls, including miR-376a-3P [21]. Notably, miR-376a directly targets *IGF1R* to downregulate the IGF1R signaling pathway and affects melanoma cell migration [22]. These findings indicated that miR-376a played a key role in regulation of bovine adipogenesis. The findings of studies using bioinformatics online tools analyses have revealed the post-transcriptional regulation of genes relevant to adipogenesis via bta-miR-376a. Investigation of the regulation of bovine preadipocytes by bta-miR-376a may improve understanding of the molecular mechanism of bovine adipocytes and may profoundly impact meat quality.

## 2. Materials and Methods

### 2.1. Ethics Statement

All animal procedures for experiments were approved by the Committee of Experimental Animal Management (EAMC) at Northwest A&F University, China (protocol number: NWAFUCAST2018–167). Moreover, the use of experimental animals was carried out in accordance with the rules and guidelines of the organization and government.

### 2.2. Isolation of Tissue Samples and Preadipocytes from Qinchuan Beef Cattle

The tissue and cell samples used in the experiment in this study were collected from three 1-day-old healthy Qinchuan beef bulls bred from the National Beef Cattle Improvement Center of Northwest Agriculture and Forestry University (Yangling, China). Surgical instruments were used to collect tissue samples including heart, liver, spleen, lung, and muscle. The samples were placed in sterile, DNase- and RNase-free cell cryotubes and immediately frozen in liquid nitrogen. The samples were then stored in a freezer at −80 °C for later use.

We used one-day-old healthy bulls to isolate the original bovine pre-adipocytes, removed the adipose tissue under aseptic conditions, washed with phosphate buffer saline(PBS) supplemented with 10% antibiotics (penicillin/streptomycin) 3 times, and then added Ⅰ Collagenase digestion for 1–2 h in a 37 °C water bath shaker. Following digestion, we filtered with a cell sieve, and discarded the supernatant after centrifugation. Red blood cell lysate was added for lysis, and then an appropriate amount of DMEM-F12 (Gibco, Grand Island, NY, USA) medium containing 10% fetal bovine serum (FBS, Invitrogen, Waltham, MA, USA) was added to resuspend the cells for seeding for subsequent experiments. The cells used in this experiment were all the third generation cells after passage.

### 2.3. Cells Transfection

In this study, bovine preadipocytes and HEK293A cells (Laboratory reservation) were cultured in DMEM-F12 and DMEM high-glucose medium (Gibco, Grand Island, NY, USA) and at 37 °C with 5% CO_2_, respectively. According to the transfection scheme of liposome 3000 (Invitrogen, Waltham, MA, USA) [8], bta-miR-376a mimic (50 nM), mimic NC (50 nm), si-KLF15 (100 nM), and the si-NC were transfected into bovine preadipocytes. The sequences of si-KLF15 are as follows: Sense 5′-CUGGAGGAGAUUGAAGAGUTT-3′ and antisense 5′-ACUCUUCAAUCUCCUCCAGTT-3′. The transfection concentration was 80%, 0.5 mm 3-Isobutyi-1-methylxanthine (IBMX), and 1 μm DXMS and 2 μm insulin were used to stimulate the differentiation of the fusion cells. Bta-miR-376a mimic and mimic NC used in this stage were purchased from RiboBio, Guangzhou, China, si-KLF15 and si-NC used in this stage were purchased from GenePharma, Shanghai, China.

### 2.4. Western Blotting Analysis

Protein was obtained from bovine preadipocytes using a protein extraction kit, supplemented with PMSF (Beyotime, Shanghai, China), and protein concentration was quantified with the Quantitative Protein Kit (the BCA method) (APPLYGEN, Beijing, China). For Western blot, 20 μg total cellular protein was separated on a 10% polyacrylamide gel, and then transferred onto PVDF. After blocking in Quick Block™ Western blocking solution (Beyotime, Shanghai, China) for 30 min at RT, the PVDF was sequentially incubated overnight at 4 °C with primary antibodies, respectively. Table 1 lists the dilution factor and company name of different antibodies. After washing in TBST-1× buffer 3 times (10 min each time) (Sangon Biotech Co., Ltd., Shanghai, China), the Goat anti-rabbit HRP antibody (absin, 1:10,000) diluted in Secondary Antibody Dilution Buffer (Beyotime, Shanghai, China) was incubated for 2 h, and washed in TBST-1× buffer.

### 2.5. EdU Staining

EdU Staining was performed with a Cell-Light EdU DNA Proliferation Kit (RiboBio, Guangzhou, China). A total of 1 × 10^5^ bovine precursor cells were implanted into 24-well cell culture plates, and transfection (bta-miR-376a mimic and mimic NC) was performed when the cells increased to a density of 50–60%. After 48 h, a cell cycle detection kit was used for detection. Finally, fluorescence photography was carried out under an optical microscope. 

### 2.6. Flow CytoMetry (FCM)

Preadipocytes were cultured in a 6-well cell culture plate and then transfected with bta-miR-376a, which mimic NC when the cell density is 40–50%. After 48 h, the cells were collected and then added to 250 μL DNA staining solution and 2.5 μL osmotic solution (Multisciences, Hangzhou, China). Flow cytometry (FCM II, BD Biosciences, San Jose, CA, USA) was used to analyze the cell cycle and count 10,000 cells.

### 2.7. CCK-8 Assay

Preadipocytes were cultured in 96-well plates at a cell density of 1 × 10^4^ cells/well. Cell proliferation was detected after 48 h of transfection using the Trans Detect Cell Counting Kit (CCK) (TransGen Biotech Ltd., Beijing, China). Thereafter, CCK solution (10 μL) was added to wells, and the plates were incubated at 37 °C for 4 h under 5% CO_2_ atmosphere. 

### 2.8. Oil Red O Staining and Triacylglycerol Assay

The cells were washed with PBS three times, then fixed in 4% paraformaldehyde for 30 min, then stained with 60% oil red O (solvent: isopropanol, 0.15 g oil red powder/100 mL), and kept away from light for 30 min, then washed with PBS and examined under a microscope. The samples were added and reacted at 37 °C or 25 °C for 15 min. The color was stable within 60 min after the reaction was balanced. The standard curve was drawn to calculate the triglyceride concentration. Finally, the triglyceride content per mg protein concentration was corrected. Specific steps can be found in the protocol [23].

### 2.9. Luciferase Reporter Assay

To verify whether miR-376a targets the 3‘-UTR of KLF15, we obtained the mature sequence of bta-miR-376a from the miRBase (http://www.mirbase.org/). The reporter of luciferase was produced by ShengGong (Sangon Biotech Co., Ltd., Shanghai, China) through synthesizing 3′-UTR of KLF15 wild-type (WT) and mutation-type (MUT) of KLF15 sequence. Xhol and Notl cutting sites of WT3′-UTR containing miR-376a target were amplified. KLF15-3′UTR WT or MUT was cloned into psiCHECK-2 vector (Laboratory retention) by Xhol and Notl restriction sites (Promega, Madison, WI, USA). HEK293A cells were cultured for two days, and then transfection experiments were performed. Approximately 0.16 μg of MUT vector/KLF15-3′UTR WT was co-transfected into HEK393A cells with five pmol miR-376a mimic/negative control. At 48 h after transfection, the luciferase activity was measured by the Dual-Luciferase^®^ Reporter Assay System (Promega, Madison, WI, USA). The luciferase assay was carried out in three repeat wells and three experiments were carried out.

### 2.10. Statistical Analysis

Data are expressed as mean ± standard error of the mean (SEM). Statistical analysis was performed using SPSS 20.0 Statistics software (SPSS, IBM Corp., Endicott, NY, USA, 2011) and GraphPad Prism 6 [24]. Independent t-tests were used to compare the means of the two groups. There are three replicates for each experiment. Differences between the means were considered to be statistically significant when * *p* ≤ 0.05, ** *p* ≤ 0.01, and *** *p* ≤ 0.001.

## 3. Results

### 3.1. The Profile of Bta-miR-376a in Adipogenesis

**Figure 1:**
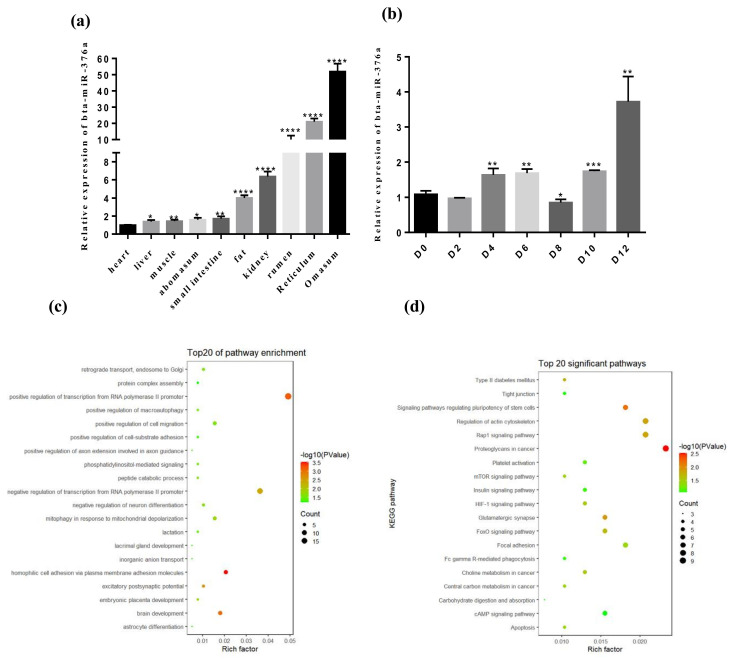
The specific changes of bta-miR-376a in tissue and time were observed. (**a**) Expression profile of bta-miR-376a in different tissues. (**b**) Expression of bta-miR-376a in bovine adipocytes at 0, 2, 4, 6, 8, 10, and 12 days of differentiation. (**c**) Gene Ontology (GO) analysis of bta-miR-376a target gene predicted. (**d**) Analysis of Kyoto Encyclopedia of Genes and Genomes (KEGG) pathway of bta-miR-376a target genes predicted. The above results represent the mean ± standard error of the mean (SEM) of three replicates (*n* = 3). * *p* ≤ 0.05, ** *p* ≤ 0.01, *** *p* ≤ 0.001, and **** *p* ≤ 0.0001.

We analyzed the expression of bta-miR-376a by RT-qPCR to determine the role of bta-miR-376a in adipocyte development. The bta-miR-376a levels were higher in the stomach and reticular tissues than those in the heart, liver, and adipose tissues (Figure 1a). We then investigated bta-miR-376a expression in preadipocytes from Qinchuan beef cattle. We observed an upward trend of bta-miR-376a levels during the preliminary stage, which then decreased during the differentiation process. The expression levels of bta-miR-376a increased after day 8 (Figure 1b). The results of Gene Ontology(GO) analyses of possible target genes of bta-miR-376a indicated that bta-miR-376a was involved in the development of adipocytes, especially proliferation and differentiation (Figure 1c). Among the first 20 important Kyoto Encyclopedia of Genes and Genomes (KEGG) pathways analyzed, many were associated with the biological processes of adipocytes (Figure 1d). Therefore, we considered that bta-miR-376a would play a crucial role in adipogenesis in Qinchuan beef cattle.

### 3.2. Bta-miR-376a Inhibits the Proliferation of Bovine Preadipocytes

**Figure 2:**
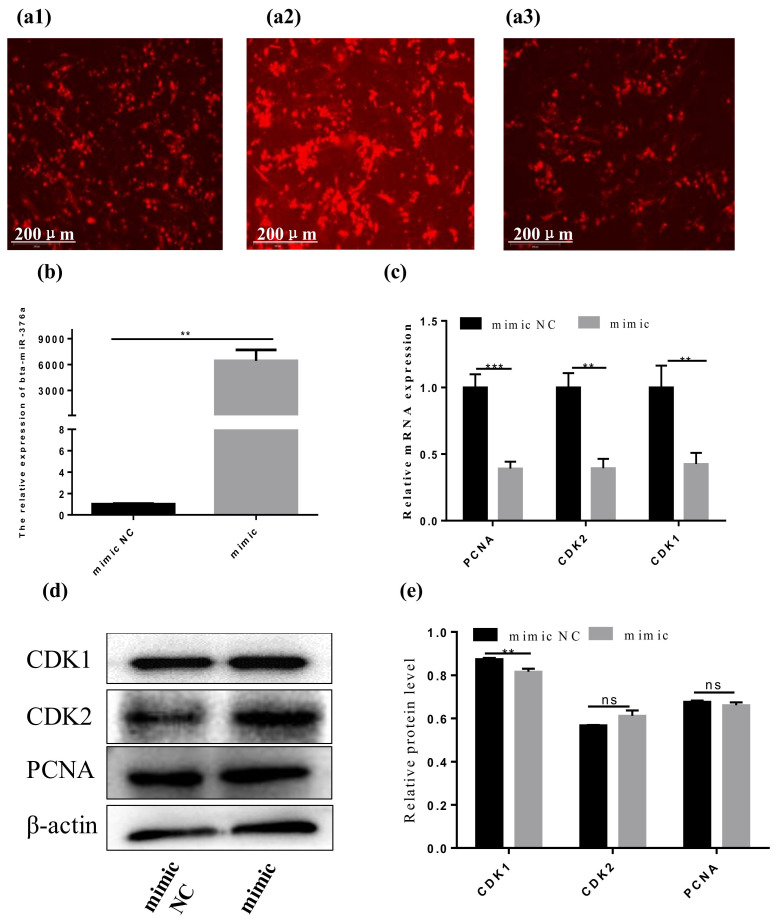

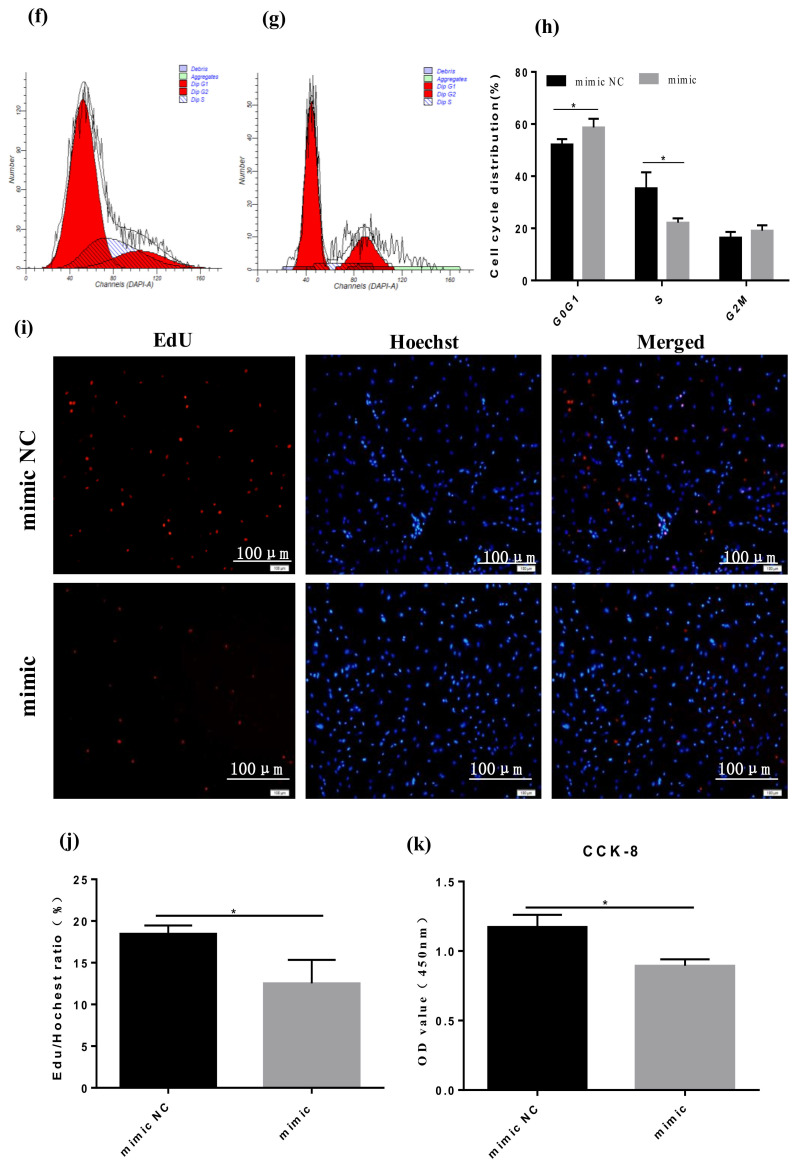
Bta-miR-376a inhibited the proliferation of Qinchuan beef preadipocytes. (**a1**–**a3**) Using the evos-fl-auto2 automatic living cell fluorescence microscope imaging system (Thermo Fisher, Waltham, MA, USA) to collect Cy3 fluorescence for 12, 24, and 48 h to detect the transfection status of the entire cell experiment and ensure that the transfection reagent is effective. (**b**) Quantified results of transfection efficiency detection of bta-miR-376a. (**c**–**e**) RT-qPCR and Western blot were used to analyze the expression of genes (*PCNA*, *CDK1*, *CDK2*) which associated with cell cycle. (**f**–**h**) Flow cytometry was used to analyze the cells 48 h after transfection, and the cell cycle analysis charts of different cell cycles were compared. (**i**, **j**) EdU assay was performed 48 h after transfection. Red: EdU (Cells during DNA replication); blue: Hoechst (the cell nuclei). (**k**) The amount of cells was analyzed by CCK-8. Data are expressed as mean ± SEM, * *p* ≤ 0.05, ** *p* ≤ 0.01, *** *p* ≤ 0.001.

We transfected bta-miR-376a mimic and the negative miRNA control mimic (NC) into Qinchuan beef cattle preadipocytes. We simulated NC with CY3 fluorescence emission in cells at different times to verify that transfection had proceeded normally (Figure 2a1–a3), and then detected bta-miR-376a levels to determine transfection efficiency. Expression levels of bta-miR-376a increased more than 6000 times compared to NC (*p* ≤ 0.01) (Figure 2b), indicating efficient transfection. The bta-miR-376a mimic significantly (*p* ≤ 0.01) inhibited the expression of cell cycle-related genes at the mRNA and protein levels (Figure 2c–e). Upregulation of the bta-miR-376a mimic lowered the ratio (%) of S-phase cells (Figure 2f–h). We then analyzed the function of bta-miR-376a in preadipocyte proliferation using EdU staining and cell proliferation kits. Figure 2i,j shows that the overexpressed bta-miR-376a mimic evidently reduced the number of 5-ethynyl-2′-deoxyuridine (EdU)-labeled cells. The CCK-8 findings showed less proliferation of adipocytes transfected with the bta-miR-376a mimic than NC (Figure 2k). These results confirmed that bta-miR-376a inhibited adipocyte proliferation. 

### 3.3. Bta-miR-376a Represses Adipocyte Differentiation

**Figure 3:**
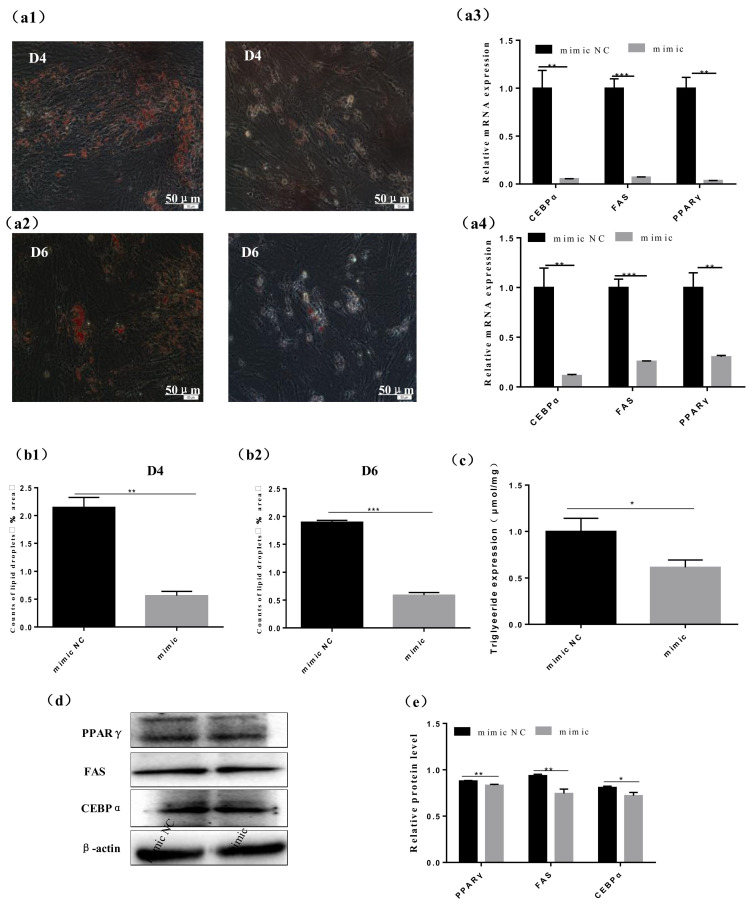
Effect of Bta-miR-376a on the morphology and gene expression of differentiated bovine adipocytes. (**a1**–**a4**) Oil red O staining was performed on the bovine adipocytes on the d4 and d6 of differentiation. The relative mRNA expression of adipogenic genes in the bovine adipocytes on the fourth and sixth day of differentiation was used by Olympus Image taken by IX71 microscope (Olympus, Japan). (**b1**, **b2**) Image J software was used to count lipid droplets in bovine adipocytes on d4 and d6 of differentiation. (**c**) The content of triglyceride was detected at 550 nm. (**d**) The adipogenesis genes of the bovine adipocytes transfected with bta-miR-376a mimic and mimic NC on d4 of differentiation were analyzed by Western blot. (**e**) The quantitative analysis of PPARγ, FAS, and CEBPα was performed by Western blotting. Data are expressed as mean ± SEM, * *p* ≤ 0.05, ** *p* ≤ 0.01, *** *p* ≤ 0.001.

We investigated the function of bta-miR-376a in the differentiation of adipocytes from Qinchuan beef cattle. We found active bta-miR-376a expression during the proliferation stage of bovine preadipocytes (*p* ≤ 0.001), which gradually decreased until day 6 (metaphase differentiation), reached the nadir on day 8, and then began to increase (Figure 1b), reaching a peak at the completion of adipocyte differentiation on day 12. We evaluated the expression of adipogenic markers at the mRNA and protein levels to elucidate the roles of bta-miR-376a in the differentiation of Qinchuan beef cattle adipocytes. Figure 3a3,a4,d,e shows that bta-miR-376a significantly decreased levels of CEBPα, PPARγ, and FAS gene expression (*p* ≤ 0.05 and *p* ≤ 0.01). Triglyceride contents were higher in the NC than the bta-miR-376a mimic group (Figure 3c). Staining with oil red O also confirmed that bta-miR-376a overexpression during differentiation could reduce lipid droplets in bovine adipocytes (Figure 3a1,a2,b1,b2). These results indicated that bta-miR-376a was a negative regulator of Qinchuan beef cattle adipocyte differentiation.

### 3.4. Bta-miR-376a Affects Qinchuan Beef Cattle Preadipocyte Differentiation by Targeting KLF15 and by Regulating the Adipogenesis Signaling Pathway

**Figure 4:**
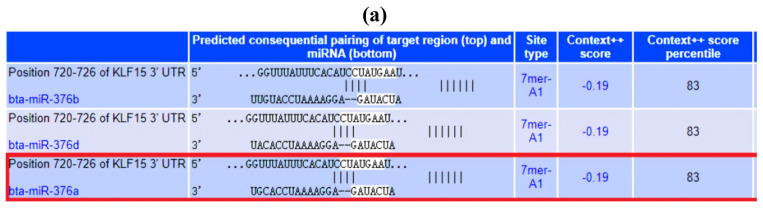

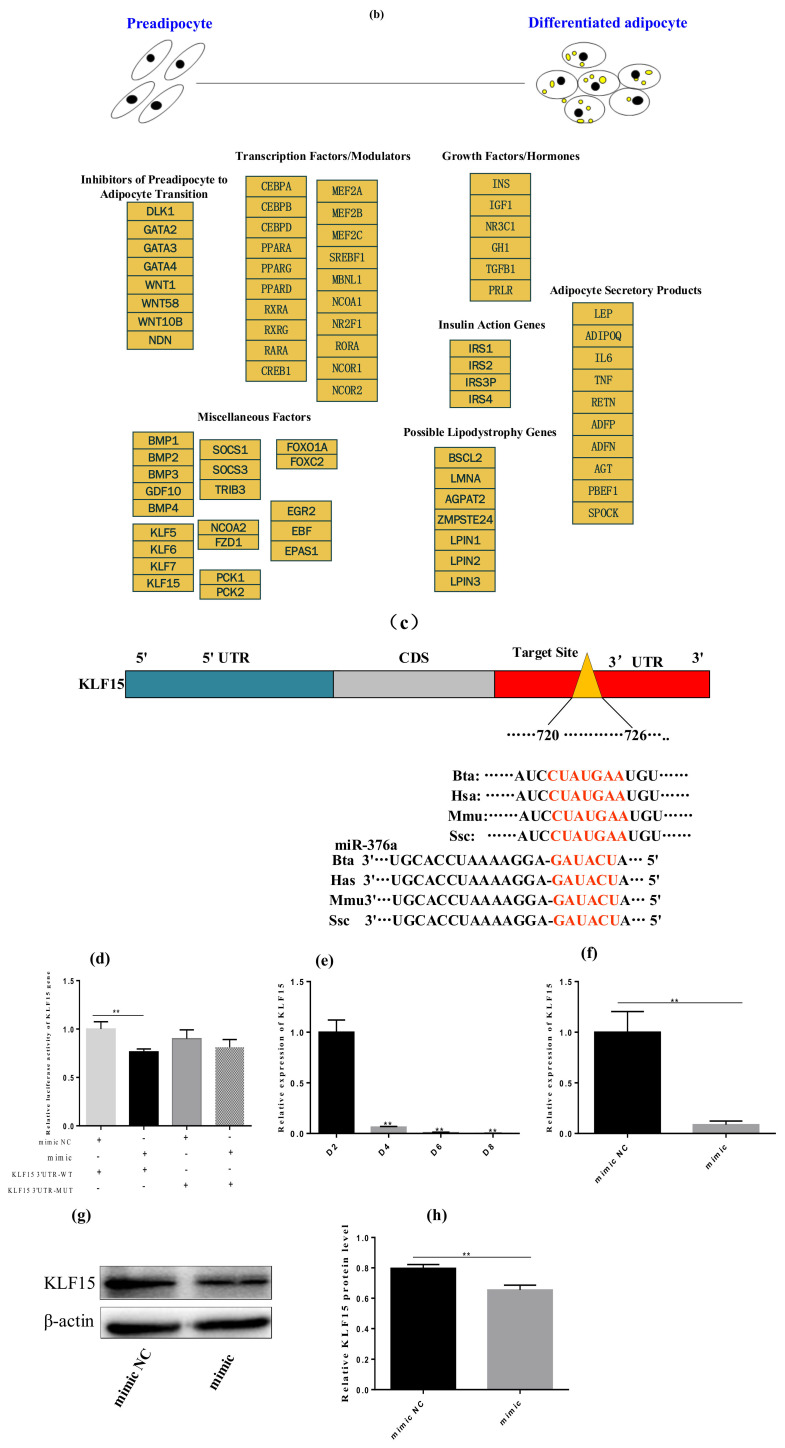
(**a**) *KLF15* is a target gene of bta-miR-376a in the differentiation of Qinchuan beef preadipocytes. (**b**) Adipogenesis signaling pathway. (**c**) Sequence alignment of bta-miR-376a in 3′UTR of Bta (cattle), Human (HSA), Mouse (MMU), and Pig (SSC). (**d**) Changes of luciferase activity after co-transfection. (**e**) KLF15 expression during adipogenic differentiation at the mRNA level. (**f**) The relative expression of KLF15 was observed after treatment. (**g**) The expression of KLF15 protein was analyzed by Western blot after transfection of bta-miR-376a mimic and bta-miR-376a mimic NC. (**h**) KLF15 protein level was quantified. Data are expressed as mean ± SEM, ** *p* ≤ 0.01.

We used Targetscan 7.1 (http://www.targetscan.org/vert_71/) to predict the potential target gene of bta-miR-376a and to elucidate the mechanism by which it regulated adipogenesis (Figure 4a). We selected the significant adipocyte differentiation-related gene, Krüppel-like transcription factor 15 (KLF15), as a candidate target gene. We found that two super pathways were associated with KLF15 in GeneCards (https://www.genecards.org/), and that one pathway was responsible for the adipogenesis effect (Figure 4b). We constructed luciferase reporter vectors of the wild-type KLF15 3′UTR and mutant KLF15 3′UTR (Figure 4c). Dual-luciferase reporter assays showed that KLF15 was a target gene of bta-miR-376a (Figure 4d). To further confirm the association between bta-miR-376a and KLF15, we assessed KLF15 expression during adipogenic differentiation at the mRNA and protein levels and found opposing trends of KLF15 and bta-miR-376a, especially during the early stages of differentiation (Figure 4e–h). Overall, these findings indicated that bta-miR-376a promoted adipocyte differentiation and regulated adipogenesis signal transduction pathways by directly targeting KLF15.

### 3.5. siRNA-KLF15 Reduces Triglyceride Content

**Figure 5:**
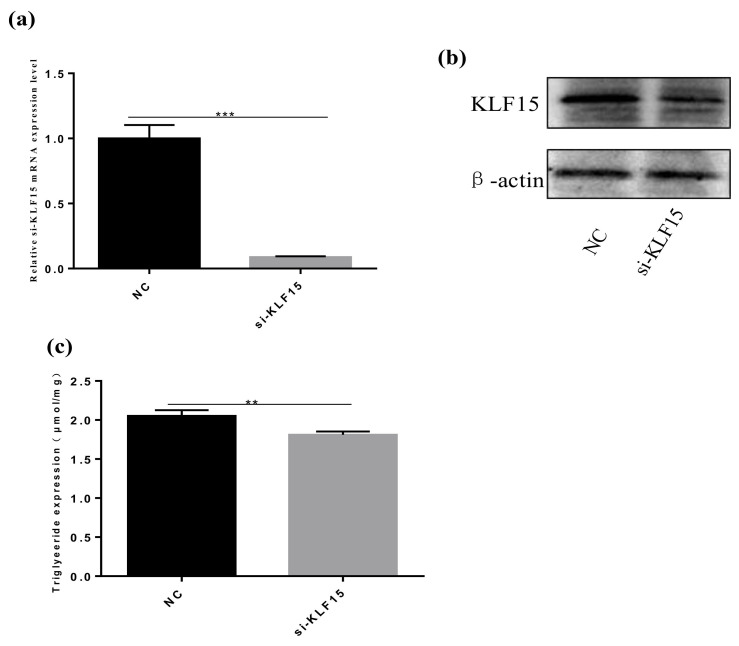
Effects of KLF15 on the metabolism of adipocytes in Qinchuan cattle. (**a**, **b**) The expression of KLF15 mRNA and protein in preadipocytes transfected with si-KLF15 and negative control cells were compared. (**c**) After si-KLF15 treatment, the triglyceride level of preadipocytes decreased. ** *p* ≤ 0.01, *** *p* ≤ 0.001.

We used si-KLF15 interference to determine the role of KLF15 in pre-adipocytes from Qinchuan beef cattle. We determined the optimal siRNA concentration required to ensure high knockout efficiency before transfection (Figure 5a,b), and then found that silencing the KLF15 gene with 100 nM si-KLF15 reduced triacylglycerol (TAG) levels in adipocyte precursors, indicating that KLF15 was a key transcription factor in pre-adipocytes from Qinchuan beef cattle (Figure 5c).

## 4. Discussion

Qinchuan beef cattle are an excellent local cattle breed in China and the first of China’s five major cattle breeds. It has a large physique and good meat production performance. It is named after the “eight hundred miles of Qinchuan” produced in the Guanzhong region of Shaanxi Province [25]. Compared with other cattle, it has the advantages of tender meat, rich meat taste, and good marble pattern, but it has a slow growth rate, low meat production, and lack of fat deposition [26]. This study intended to reduce bovine body fat deposition and increase intramuscular fat deposition through molecular level regulation, which is expected to solve the problems of slow growth, low meat production, and insufficient fat deposition in Qinchuan beef cattle [27].

The present study demonstrated that bta-miR-376a played an important role in the regulation of preadipocyte proliferation and differentiation in Qinchuan beef cattle, as a bta-miR-376a mimic inhibited the proliferation and differentiation. Therefore, bta-miR-376a might be a crucial negative regulator in adipogenesis. Poor meat quality faced by low IMF and obesity are two major problems that should be considered from the viewpoints of animal science and human health [28]. Thus, we investigated the regulation of adipogenesis, which is rather complex. Strictly controlled cell regulation along with the participation of many transcription factors and signal pathways pose challenges in the elucidation of mechanisms of adipogenesis [29,30]. Many regulatory factors associated with adipogenesis, as well as regulatory methods of epigenetics and miRNA, have been identified [28,29,30,31]. Several novel miRNA have been identified using microarray analysis and high-throughput sequencing [32]: miR-424 [33], miR-210 [34], miR-26a [35], miR-17-5p [36], miR-152 [37], miR-143 [38], and miR-519d [39] reduce adipogenesis by positively facilitating adipocyte differentiation, whereas miR-20a-5p [40], miR-130a/b [23], miR-27a/b [41,42], and miR-155 [43] negatively regulate adipocyte differentiation. The expression of bta-miR-376a differs among bovine tissues and is tissue-specific. High bta-miR-376a levels were expressed during the precursor adipocyte proliferation phase (before induction of differentiation), and were maximal in fully mature differentiated adipocytes at 12 days, suggesting that bta-miR-376a simultaneously regulated the proliferation and differentiation of bovine adipocytes. Bta-miR-149-5p inhibits the proliferation and differentiation of preadipocytes in Qinchuan beef cattle [8], and miR-30a-3p simultaneously inhibits the proliferation and differentiation of muscle cells [44]. The data obtained from the analysis of online bioinformatics tools have predicted a relationship between bta-miR-376a and adipocyte metabolism. We determined the role of bta-miR-376a in adipocyte proliferation and differentiation and confirmed that bta-miR-376a was a potential negative regulator of adipocyte formation. These results provide a new approach to tackle the issue of low IMF content in terms of breeding Qinchuan beef cattle. The data obtained from the analysis of online bioinformatics tools have also shown a higher bta-miR-376a concentration in pathways associated with adipocyte metabolism. Others have shown that KLF plays diverse roles during mammalian cell differentiation and development [45]. The first KLF discovered during adipogenesis was KLF15 [46], implying that KLF15 might be necessary for adipocyte biology and essential regulation of lipid renewal in adipocytes. Indeed, KLF15 is a downstream effector of C/EBPβ and PPARγ, which can induce white adipocyte differentiation [47], and it activates the closest GGGG element located in the -264–76 region of the bovine KLF3 promoter, and binds to the core promoter region of KLF3 in preadipocytes from Qinchuan beef cattle. These findings suggest that KLF15 promotes the transcriptional activity of KLF3 in adipocytes from these cattle [48]. 

All of these findings confirmed our previous work. The “CUAUGAA” of 3′UTTR of the KLF15 gene sequence can combine with the seed sequence of bta-miR-376a. When bta-miR-376a is overexpressed, the target gene KLF15 is downregulated. PPARγ is a downstream effector of KLF15 in adipocyte differentiation. A decrease in KLF15 expression reduces the activity of the PPARγ combination. PPARγ plays a pivotal role in adipogenesis. On the one hand, it can promote the expression of CEBPα, and its expression also requires the participation of CEBPα. This is why CEBPα decreases when overexpressing bta-miR-376a. FAS is a key enzyme in acetyl-coA and malonyl coA synthesis of triglycerides. When KLF15 is downregulated, it inhibits fat cell maturation and interferes with processes that regulate glucose production (mainly gluconeogenesis and amino acid decomposition). Therefore, the FAS gene is downregulated as well. Big data shows (Pathcards database) that the lipogenic signaling pathway is one of the important pathways in which KLF15 mainly participates. Therefore, genes in this pathway or other genes related to PPARγ will also change to varying degrees. Just like the signal transduction process, a stimulus at one point will lead to a series of biological processes, and then produce changes in phenotype. When we interfered with the KLF15 gene, compared with the NC group, the triglyceride content in adipocytes was indeed downregulated, which confirmed the positive role of KLF15 in the process of adipogenic differentiation. These findings provide the basis for further studies on the bta-miR-376a-KLF15-PPARγ pathway. 

## 5. Conclusions

The novel regulatory factor, bta-miR-376a, affected the growth and development of Qinchuan beef cattle. It negatively regulated the proliferation and differentiation of adipocytes from these cattle via targeting of the adipogenesis signaling pathway. These findings contribute to a better understanding of miRNA regulation of adipogenesis and provide an important reference for future studies on beef IMF. Further studies should investigate the bta-miR-376a-KLF15-PPARγ pathway to deepen the understanding of the role of miRNA regulation of adipogenesis.

## Figures and Tables

**Table 1:** Antibody information.

Name of Antibody	Immune Features	Company	Dilution Ratio
anti-KLF15 antibody	Rabbit Polyclonal	Novus	1:300
anti-CDK1 antibody	Rabbit monoclonal	Abcam	1:1000
anti-CDK2 antibody	Rabbit monoclonal	Abcam	1:1000
anti-PCNA antibody	Rabbit monoclonal	Abcam	1:5000
anti-PPARγ antibody	Rabbit Polyclonal	Boster	1:1000
anti-FAS antibody	Rabbit monoclonal	Abcam	1:5000
anti-CEBPα antibody	Rabbit monoclonal	Abcam	1:1000
anti-β-actin antibody	Rabbit Polyclonal	Abcam	1:10,000
